# Cross-Link Guided Molecular Modeling with ROSETTA

**DOI:** 10.1371/journal.pone.0073411

**Published:** 2013-09-17

**Authors:** Abdullah Kahraman, Franz Herzog, Alexander Leitner, George Rosenberger, Ruedi Aebersold, Lars Malmström

**Affiliations:** 1 Department of Biology, Institute of Molecular Systems Biology, Eidgenössische Technische Hochschule Zürich, Zurich, Switzerland; 2 Gene Center, Ludwig-Maximilians-Universität München, Munich, Germany; 3 Faculty of Science, University of Zurich, Zurich, Switzerland; Aberystwyth University, United Kingdom

## Abstract

Chemical cross-links identified by mass spectrometry generate distance restraints that reveal low-resolution structural information on proteins and protein complexes. The technology to reliably generate such data has become mature and robust enough to shift the focus to the question of how these distance restraints can be best integrated into molecular modeling calculations. Here, we introduce three workflows for incorporating distance restraints generated by chemical cross-linking and mass spectrometry into *ROSETTA* protocols for comparative and *de novo* modeling and protein-protein docking. We demonstrate that the cross-link validation and visualization software *Xwalk* facilitates successful cross-link data integration. Besides the protocols we introduce *XLdb*, a database of chemical cross-links from 14 different publications with 506 intra-protein and 62 inter-protein cross-links, where each cross-link can be mapped on an experimental structure from the Protein Data Bank. Finally, we demonstrate on a protein-protein docking reference data set the impact of virtual cross-links on protein docking calculations and show that an inter-protein cross-link can reduce on average the RMSD of a docking prediction by 5.0 Å. The methods and results presented here provide guidelines for the effective integration of chemical cross-link data in molecular modeling calculations and should advance the structural analysis of particularly large and transient protein complexes via hybrid structural biology methods.

## Introduction

Conventional structural biology techniques like X-ray crystallography or Nuclear Magnetic Resonance (NMR) spectroscopy solved the structure of a large number of macromolecular complexes [1]. High-resolution data from those techniques provide detailed insights into the working principles of proteins and their malfunction [2] and support drug discovery projects [3]. However, the requirement of these techniques for relatively large amounts of pure and highly concentrated protein samples has caused a bias in the structural elucidation of monomeric proteins and homomeric protein complexes. The PISA database [4] lists around 13,600 heteromeric protein complexes compared to around 62,000 mono- and homomeric proteins as of June 2013. Structural data on large protein complexes or transient interactions in cell signaling processes have therefore remained rather elusive [5].

Chemical cross-linking in combination with mass spectrometric analysis (XL-MS) has emerged as a viable tool for probing the structure of many protein complexes without such bias. Although XL-MS only provides comparatively low resolution data [6], [7], it is highly complementary to conventional methods, has less stringent sample purity requirements and few restrictions concerning complex size. Recent applications of XL-MS have elucidated the structure and topology of RNA polymerases [8], [9], proteasomes [10], [11] and the chaperonins GroEL [12] and TRiC [13], [14]. Most often, homobifunctional cross-linking reagents, for example amine-reactive succinimide esters are used in XL-MS studies. They react predominantly with primary amine groups on lysine side chains and N-termini [15]. The read-out of such an experiment is a list of modified sites, which are obtained from the analysis of fragment ion spectra generated by the mass spectrometric analysis of cross-linked peptides. The peptides, in turn, are generated by the enzymatic digestion of a cross-linked protein complex. Reaction products can be broadly classified into mono-links, i.e. single peptides where only one end of the cross-linker has reacted, and different types of cross-linked peptides. Of particular interest are intra-protein and inter-protein cross-links that originate from connecting two peptide chains from a single or two different polypeptides, respectively, by the cross-linking reagent. Note that different nomenclatures have been proposed in the literature to describe the various types of products of cross-linking reactions [16], [17]. Recent advances in the protocols to generate and process cross-linked samples [18], [19], the mass spectrometric methods to generate fragment ion spectra of cross linked peptides [20] and the development of software tools for the identification of the cross-linked peptides [21], [22] have contributed to the increasing maturity and robustness of the XL-MS technology.

The distance restraints generated by XL-MS can be used to guide molecular modeling simulations towards native-like conformations. However, two important points need to be addressed prior to the incorporation of XL-MS distances. First, cross-linker molecules are flexible and can covalently link lysine residues over a large range of inter-residue distances [23]. In the current work, all calculations are based on data using disuccinimidyl suberate (*DSS*) as a reagent. DSS has a spacer length of approximately 11.4 Å, but was experimentally found to bridge lysine residues of up to 30.0 Å and more (calculated as Cα-Cα protein backbone carbon atom Euclidean distance). This distance restraint takes into account the length of two extended lysine side chains (∼5.5 Å each) and some conformational flexibility of the protein complex [6]. Second, cross-linker molecules can be assumed to not penetrate the protein surface and be located on solvent accessible surface patches [24]. In case cross-links are simulated as distance restraints in modeling calculations, the linear Euclidean distance measure becomes inappropriate, as it will penetrate the protein surface. Both limitations can either be solved by explicitly modeling the cross-linker molecule [25] or by implementing a non-linear distance measure [24]. We previously introduced the *Xwalk* (“Crosswalk”) algorithm [26] to calculate the shortest path between two cross-linked amino acids, where the path must not penetrate the protein surface and only lead through solvent occupied space. The algorithm is based on a cubic grid around the cross-linked amino acids, a distance calculator that fills the grid cells with distances following a breadth-first search algorithm, and a trace-back method that selects the shortest path through the grid between cross-linked amino acids. The length of the shortest path is a distance measure that we termed Solvent Accessible Surface (*SAS*) distance, which represents a more reasonable measure of cross-link distances in modeling calculations.

We recently applied the *Xwalk* algorithm in combination with the *ROSETTA* molecular modeling suite to affinity purified protein complexes from the Protein Phosphate 2A (PP2A) network [6]. The modeling calculations were guided by 176 inter-protein cross-links and 570 intra-protein cross-links. Within this study we were able to verify comparative models of all PP2A subunits, predict the location of an intrinsically disordered C-terminal domain of the PP2A interactor IgBP1, define the binding interface between IgBP1 and the catalytic phosphatase subunit, and determine the topology of the regulatory subunit 2ABG in complex with the TCP1 Ring Complex (TRiC) chaperonin.

Here we describe in detail three computational modeling workflows that we applied in the above study [6] as a hybrid structural biology method for *de novo* prediction of protein structures, comparative modeling of proteins and protein-protein docking. A demo version of the docking protocol is available in the *ROSETTA* protocol capture archive: XL_guided_protein_docking/run_demo.sh. We also introduce a database with literature-curated cross-links, which we exploited for computing probability distributions of cross-link distances. And finally, we demonstrate in a systematic study the association between the accuracy of a cross-link guided protein docking calculation and the number of employed inter-protein cross-links.

## Methods

All modeling calculations were performed using the *ROSETTA* molecular modeling suite. Protocols applied within the workflows are available in the public release of *ROSETTA* starting from version 3.1, with the exception of the protocol *nonlocal*, which was downloaded from *ROSETTA*’s trunk at revision 42791. The reader is kindly referred to the help pages of the individual software programs for a detailed explanation of the employed application parameters. Parameter arguments shown in curly brackets require case specific input.

All workflows have in common that they utilize XL-MS data to guide modeling calculations towards native-like conformations with the assumption that all identified cross-links were formed on native structures. The guidance was achieved by two means. Firstly, by incorporating the cross-link distance restraints into the ROSETTA scoring function where they penalize models with cross-link distances above the distance threshold of 30.0 Å, which leads to a preferential sampling of the conformational space around the native conformation. Secondly, by applying XL-MS distances as post-modeling filters in the candidate selection stage, where they removed models that violate cross-link data.

### Comparative Modeling

**Figure 1:**
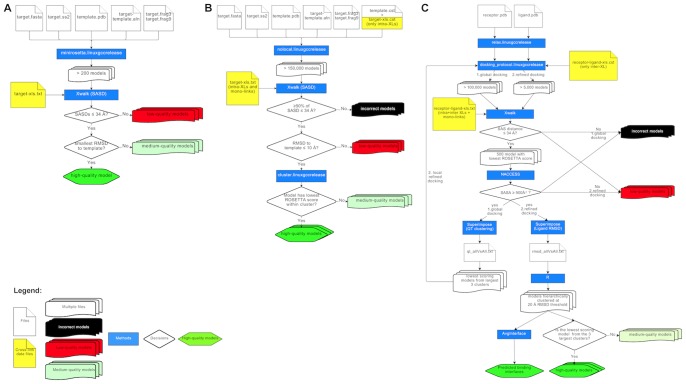
Computational workflows for cross-link guided molecular modeling centered on *ROSETTA* protocols and *Xwalk* software. (A) Comparative modeling. (B) *De novo* modeling with partial structural information. (C) Protein-protein docking. Flowcharts were generated using https://www.draw.io.

The structure of a target protein can be predicted with comparative modeling, if experimental X-ray crystallography or NMR structures of a homologous template protein exist. Out of the 94 proteins that were purified from the PP2A interaction network [6], only 8 (2AAA_HUMAN, 2A5G_HUMAN, 2ABA_HUMAN, PP2AA_HUMAN, SGOL1_HUMAN, SET_HUMAN, MST4_HUMAN, DYL1_HUMAN) had partial or complete structural information from X-ray crystallography experiments. For a subset of 15 proteins comprising all remaining PP2A core subunits and the eight TRiC chaperonin proteins, high quality comparative models were generated using various homologous template structures (see Table 1) and the following workflow (see also Figure 1A). The typical execution time of the workflow is about 5 CPU days per protein.

**Table 1:** Overview of 15 proteins from the PP2A network for which comparative models were generated.

UniProt Entry name(_HUMAN)	Template PDB-ID –Chain ID	SequenceIdentity [%]	Number ofexperimental XLs	Number ofsatisfied XLs	Min RMSD	Min RMSDwith XLs	Score of minRMSD with XLs	Rank of minRMSD with XLs
TCPB	2XSM-A	98.1	30	28	0.9	0.9	−2024.8	3
TCPD	2XSM-B	96.5	9	9	0.8	1.0	−2006.8	3
PP2AB	3FGA-C	95.1	1	1	1.2	1.2	−597.0	1
2A5D	3FGA-B	59.5	11	11	1.3	1.3	−1385.5	1
2A5E	3FGA-B	62.8	11	11	1.5	1.5	−1393.1	1
2A5A	3FGA-B	60.0	6	6	1.5	1.5	−1396.4	1
TCPG	2XSM-D	98.5	21	21	1.6	1.6	−2184.2	2
TCPZ	2XSM-E	97.2	16	16	1.6	1.7	−2028.5	3
TCPA	2XSM-C	97.1	6	6	2.0	2.0	−2321.1	1
2ABD	3DW8-B	83.7	1	1	2.0	2.0	−937.3	1
2AAB	3FGA-A	83.9	18	13	1.3	2.3	−1192.2	139
TCPH	2XSM-G	98.2	15	15	2.0	2.4	−2070.9	3
TCPE	2XSM-H	97.4	16	16	2.5	2.5	−2226.8	186
TCPQ	2XSM-F	97.6	7	7	2.5	2.5	−2057.1	1
2ABG	2ABA-B	77.7	15	13	2.2	19.6	−544.4	124
Median					1.6	1.7		2

Listed next to the protein UniProt entry names are information on the template PDB structure, the sequence identity between the target and template protein sequences, the number of experimental cross-links collected for each protein, the largest number of cross-links that were satisfied by each protein’s best model, the minimum RMSD value to the template PDB structure observed during entire simulation, the RMSD value of the model that satisfied most cross-links while having the lowest RMSD value, the score and the rank position of that model.

- 1.1 Run HHpred [27] (http://toolkit.tuebingen.mpg.de/hhpred/) on the target sequence to determine the best template structure.
- 1.2 Download the template structure from the Protein Data Bank(http://www.pdb.org).
- 1.3 Extract the sequence alignment between target and template protein from the HHpred output. Note, that the template sequence in HHpred corresponds to the SEQRES sequence and not to the ATOM coordinate sequence. In cases in which the template structure is predetermined and has a high sequence identity to the target protein, as was the case for the PP2A and TRiC proteins, one can also use the global sequence alignment application needle from the EMBOSS package v6.2.0 [28] with default command-line flags.
- 1.4 Generate fragment files for the target protein using the ROBETTA server [29] (http://robetta.bakerlab.org).
- 1.5 Predict the secondary structure of the target protein using PSIPRED [30] (http://bioinf.cs.ucl.ac.uk/psipred).
- 1.6 Using the input files from above, run ROSETTA’s minirosetta application on each target protein using following command line flags:
  -database {rosetta_DB_dir}
  -run:protocol threading
  -run:shuffle
  -in:file:fasta {protein.fasta}
  -in:file:psipred_ss2 {protein.ss2}
  -in:file:template_pdb {template.pdb}
  -in:file:alignment {protein-template.aln}
  -out:overwrite
  -out:nstruct 200
  -out:shuffle_nstruct 200
  -cm:aln_format general
  -idealize_after_loop_close
  -out:file:silent_struct_type binary
  -loops:extended
  -loops:build_initial
  -loops:remodel quick_ccd
  -loops:relax relax
  -loops:frag_sizes 9 3 1
  -loops:frag_files {fragments9} {fragments3} none
  -frag9 {fragments9}
  -frag3 {fragments3}
  -relax:fast
  -relax:default_repeats 2
  -silent_decoytime
  -random_grow_loops_by 4
  -select_best_loop_from 1
  -in:detect_disulf false
  -fail_on_bad_hbond false
- 1.7 Predict at least 200 models per target.
- 1.8 Create a tab delimited text file (xls.txt) holding the list of all intra-protein cross-links and mono-links in a *Xwalk* specific distance file format (see also http://www.xwalk.org/cgi-bin/help.cgi#vXLtable):
  - 1 protein.pdb LYS-1-C-CBLYS-2-C-CB

where the 1^st^ column is an incremental index, the 2^nd^ column is the PDB file name and the 3^rd^ and 4^th^ columns are dash delimited PDB information about first and second cross-linked atom, respectively. The PDB information should list the residue name, residue number, chain ID and atom name. Mono-links are described with the first three columns only.

- 1.9 Calculate the SAS distance for each intra-protein cross-link using *Xwalk* [26] (http://www.xwalk.org) on each model with following command

$> java Xwalk -infile {model.pdb} -dist {xls.txt} –bb –radius 2.0–mono.

- 1.10 Choose models that satisfy the largest number of intra-protein cross-links and mono-links.
- 1.11 If multiple models satisfy the same number of cross-links and mono-links, or if none of the models satisfy any cross-link, select the model with the lowest Root Mean Square Deviation (RMSD) to the template structure as the best model.

Alternatively, distance information from XL-MS data can also be exploited as distance restraints within the scoring function (see next section), which for the PP2A project was omitted to keep the modeling unbiased for validation purposes.

### De Novo Modeling

If experimental structures are missing for a target protein and its homologs, the structure of the target protein can be predicted from its primary amino acid sequence using *de novo* modeling. The human IgBP1 protein had no crystal structure available in the Protein Data Bank (*PDB*) [31]. Structural coordinates existed only for the N-terminal domain from a mouse homolog (PDB-ID: 3CQ1). To generate a full-length model of IgBP1, we applied the following *de novo* modeling workflow (see also Figure 1B), which required about 14 CPU years of computation.

- 2.1 Generate fragment, *PSIPRED*, alignment files as described in the comparative modeling workflow above.
- 2.2 In addition, create a *ROSETTA* constraints file holding the list of intra-protein cross-link information in the following format (see also http://www.rosettacommons.org/manuals/archive/rosetta3.4_user_guide/de/d50/constraint_file.html):
  AtomPair {atom name1} {residue number1, chain ID1} {atom name2} {residue number2, chain ID2} FLAT_HARMONIC {x0} {standard deviation} {tolerance}

The flat harmonic function guarantees that models are penalized only if the Euclidean distance between two cross-linked atoms exceeds 30.0 Å. The function takes the Euclidean distance *dist* and three parameters that for a DSS cross-link were heuristically chosen to be *x_0_ = *15.0, *tolerance* = 15.0 and standard deviation *σ = *1.0:
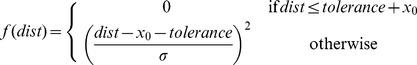

- 2.3 Run *ROSETTA*’s nonlocal application with the input files described above and the following command line flags:
  -database {rosetta_DB_dir}
  -in:file:fasta {protein.fasta}
  -in:file:psipred_ss2 {protein.ss2}
  -in:file:template_pdb {template.pdb}
  -in:file:alignment {protein-template.aln}
  -out:overwrite
  -out:nstruct {n}
  -cm:aln_format general
  -frag3 {fragments3}
  -frag9 {fragments9}
  -abinitio::relax
  -abinitio::no_write_failures
  -abinitio:increase_cycles 1
  -abinitio:rg_reweight 0.25
  -nonlocal:builder star
  -nonlocal:mode semirigid
  -nonlocal:gap_sampling_extension 5
  -jumps:ramp_chainbreaks
  -jumps:overlap_chainbreak
  -jumps:increase_chainbreak 0.5
  -constraints:cst_fa_file {template.cst}
  -constraints:cst_file {template.cst}
- 2.4 Generate at least 100,000 models.
- 2.5 Generate a *Xwalk* input file listing all cross-links and mono-links (see step 1.8) and run *Xwalk* (see step 1.9).
- 2.6 Choose top models as described in step 1.10. Choose the lowest scoring 500 models if more than 500 models should satisfy the largest number of cross-links.
- 2.7 Calculate an all against all Cα coordinate RMSD matrix among the top models.
- 2.8 Perform a hierarchical clustering with the complete clustering method using *R* [32] and the following *R* script:
  - rmsd.dat<-read.table(“rmsd_allVsAll.txt”, row.names = 1)
  - rmsd.dist<-as.dist(rmsd.dat)
  - rmsd.hist = hclust(rmsd.dist)
- 2.9 Cut the hierarchical cluster tree at an RMSD threshold value of 10 Å in the same *R* script:
  - rmsd.cut<-cutree(rmsd.hist, h = 10.0)
  - write.table(rmsd.cut, file = ”rmsd_cluster.txt”, quote = FALSE, col.names = FALSE, row.names = TRUE, sep = “\t”)
- 2.10 Pick the lowest scoring model from the largest cluster as the best model. If the clustering should retrieve only singletons, pick models that have the smallest RMSD value compared to the template structure as the best models.

### Protein-protein Docking

The catalytic subunits of PP2A were docked against best full-length models of IgBP1 (see previous section) using the following workflow (see also Figure 1C). The workflow requires a computation time of around 100 CPU days.

- 3.1.1 Prior to docking calculations, crystal structures from the PDB (e.g. PP2AA) need to be relaxed. For relaxation, run *ROSETTA*’s relax protocol with following command line flags:
  -database {rosetta_DB_dir}
  -in:file:s {protein.pdb}
  -relax:sequence
  -constrain_relax_to_start_coords
- 3.1.2 Prepare a *ROSETTA* constraint file holding a list of all inter-protein cross-links (see step 2.2).
- 3.1.3 Using the constraint file, run protein-protein docking calculations with *ROSETTA*’s docking_protocol application in low-resolution centroid mode using following command line flags:
  -database {rosetta_DB_dir}
  -in:file:s {protein.pdb}
  -constraints:cst_file {inter-xl.cst}
  -out:overwrite
  -out:nstruct {n}
  -out:file:o {model.pdb}
  -docking:low_res_protocol_only
  -docking:randomize1
  -docking:randomize2
  -docking:spin
  -docking:docking_centroid_outer_cycles 10
  -docking:docking_centroid_inner_cycles 50
  -docking:dock_lowres_filter 10 1
- 3.1.4 Generate at least 100,000 models.
- 3.1.5 Prepare a *Xwalk* input file that holds all intra-protein and inter-protein cross-links and mono-links (see step 1.8). Standard docking software and cross-link guided docking protocols utilize only inter-protein cross-links. But *Xwalk* ability to mimic cross-links by its shortest path and SAS distance calculation, allows it to additionally employ intra-protein cross-links and mono-links as post-docking filters. Predicted docking models that bury intra-protein cross-links or mono-links within their binding interface are thus detected by Xwalk and removed (see Discussion).
- 3.1.6 Assess the number of cross-links and mono-links satisfied by each model. To speed up calculations, apply first *Xwalk*’s Euclidean distance measure and subsequently the SAS distance measure (see step 1.9) on each model.
- 3.1.7 Select models satisfying to the highest number of cross-links. Should their number be higher than 500 or should none of the models satisfy any cross-link, select those 500 models with the lowest *ROSETTA* energy score.
- 3.1.8 Analyze the binding interface size of the selected models, which is equivalent to the buried surface area (*BSA*):

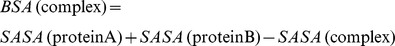
where *SASA()* is the solvent accessible surface area of the protein complex or its protein components. The SASA can be calculated with *NACCESS* [33] (http://www.bioinf.manchester.ac.uk/naccess/).

- 3.1.9 Select further only models with a sufficiently large binding interface with BSA(complex) ≥900 Å^2^ [34].
- 3.1.10 Choose a large number of models with the shortest mean SAS distance over all cross-links. (In the case of the IgBP1-PP2AA, we selected the 300 models with the shortest mean SAS distance). The shortest mean distance leads to a preference for models with overall shorter cross-link distances that in most cases show distance ranges similar to Figure S1.
- 3.1.11 Calculate an all against all Cα coordinate RMSD matrix among the top models. Note, that the RMSD should be computed only on the smaller protein (ligand) and not on the larger structure (receptor), as the latter remains fixed during the docking calculations and has an RMSD of 0 among all selected models. The RMSD among the smaller proteins is also known as the ligand RMSD (L-RMSD).
- 3.1.12 Perform a hierarchical clustering as described in step 2.8.
- 3.1.13 Cut the hierarchical cluster tree at a L-RMSD threshold value of 20 Å (see step 2.9).
- 3.1.14 Pick the lowest scoring model from the largest clusters as best models.

We would like to emphasize that the workflow described above is only one of two strategies for cross-link guided protein docking. It was developed to highlight the impact of XL-MS data on docking calculations and visualize the set of conformations that satisfy a large number of distance information from XL-MS. For a biophysically more meaningful prediction, the workflow above can alternatively be extended after step 3.1.6 with a high-resolution refinement docking stage as follows:

- 3.2.1 Run steps 3.1.1–3.1.6.
- 3.2.2 Select models that satisfy most cross-links. Should their number be higher than 500 or should none of the models satisfy any cross-link, select those 500 models with the lowest *ROSETTA* energy score.
- 3.2.3 Filter the 500 models by binding interface size (see step 3.1.8 and 3.1.9).
- 3.2.4 Apply Quality Threshold (QT) clustering on models passing the interface size filter. A QT cluster is defined by the translational and rotational distances between a reference and referred ligand structures, where the distance must be smaller than 3 Å and 8°, respectively. The distance thresholds correspond to translational and rotational perturbations that will be applied during local-refinement docking, and thus, in contrary to RMSD based clustering, will allow a seamless transition between global and local docking calculations. Rotational and translational distances can be calculated with the *Superimpose* application (http://cleftxplorer.googlecode.com/files/cX.zip), which is part of the *CleftXplorer* software package [35], [36]:
  - java –cp cX.jar:colt.jar:cdk-1.0.2.jar cX/Superimpose -dir <dir> -chain A:A -dock -xseq -ca –r –ta 3∶8

The colt.jar and cdk-1.0.2.jar *JAVA* libraries are required by the Superimpose application and can be downloaded from http://acs.lbl.gov/software/colt/colt-download/releases/colt-1.2.0.zip and http://sourceforge.net/projects/cdk/files/cdk/1.0.2/cdk-1.0.2.jar, respectively. The *Superimpose* application will read in all PDB files from the directory <dir> and output an all against all dissimilarity matrices. The dissimilarity matrix holds 0′s for similar model pairs whose translational and rotational differences are smaller than the threshold values, and 1′s for distinct models.

- 3.2.5 Complete the QT clustering by searching within the output file for the model that has the highest number of similar models (i.e. largest number of 0 in a row), declare it as the cluster representative and remove it and all its cluster members from the dissimilarity matrix. If two models have the same number of similar models, then prefer the one with the lower *ROSETTA* score. Repeat this step two additional times to obtain the cluster representatives of the largest three clusters from the global docking calculations.
- 3.2.6 Run *ROSETTA*’s docking_protocol application in high-resolution mode with 3.0 Å translational and 8° rotational Gaussian biased random perturbations [37] on the three cluster representative with a *ROSETTA* constraint file (see step 2.2):
  -database {rosetta-database}
  -in:file:s <models.pdb>
  -constraints:cst_file {inter-xl.cst}
  -docking:dock_pert 3 8
  -packing:ex1
  -packing:ex2aro
  -docking:dock_rtmin
  -docking:sc_min
  -out:nstruct {N}
- 3.2.7 Generate at least 5,000 per cluster representative
- 3.2.8 Run *Xwalk* on all models as described in step 3.1.6.
- 3.2.9 Select top 500 models as described in step 3.2.2 and 3.2.3.
- 3.2.10 Cluster models as described in step 3.1.11 to 3.1.13.
- 3.2.11 Pick the best models as described in step 3.1.14.

### Reference Data Sets for the Modeling Workflows

#### Comparative and *de novo* modeling

We have chosen 6 proteins from a recent work of Leitner and co-workers [18] for a reference data set of our comparative modeling workflow. Each protein in the data set had experimental structural data in form of an X-ray structure, a list of experimental chemical cross-links and a homologous experimental template structure. The template structures were selected such that they covered sequence identities between 50% and 90% to the native structures (see Table 2), similar to the sequence identities in the PP2A network (see Table 1).

**Table 2:** Overview of 6 proteins used as a reference data set for the comparative modeling workflow.

UniProt Entry name	PDB-ID –Chain ID	Template PDB-ID –Chain ID	Sequence Identity between UniProtand TemplateSequence [%]	Number of experimental XLs	Number of satisfied XLs	Min RMSD	Min RMSDwith XLs	Score of min RMSD with XLs	Rank of min RMSD with XLs
KPYM_RABIT	2G50-A	4IMA-A	68.5	16	12	1.3	1.4	−1145.1	1
KCRM_RABIT	1U6R-A	1QH4-A	80.8	19	18	2.2	2.2	−808.4	1
ALDOA_RABIT	3DFQ-A	1FDJ-A	71	19	9	2.4	2.4	−709.7	1
CATA_BOVIN	4BLC-A	1QQW-A	92	2	2	2.7	2.7	−1016.8	1
ALBU_BOVIN	4F5S - A	1N5U-A	75.9	55	49	3.2	3.2	−1178.2	2
TRFL_BOVIN	1BLF-A	1AIV-A	52.3	31	21	5.3	8.5	−927.7	86
Median						2.55	2.55		1

For more information regarding the columns, see Table 1.

**Note, that the RMSD values were calculated between the models and the native structure and not the template structure as in** **Table 1** **.**

One of the reference proteins, the rabbit pyruvate kinase (UniProt Entry name: KPYM_RABIT) was also chosen as a reference for the *de novo* modeling workflow. Our choice for KPYM_RABIT was due to its multidomain structure. According to the Pfam database [38], the pyruvate kinase consists of an N-terminal (amino acid positions 21–100) and C-terminal (amino acid positions 120–367) ATP:guanido phosphotransferase domain. We decided to model the former domain without any template structure and the latter domain with a creatine kinase template structure from chicken (PDB-ID: 1qh4). The sequence of the pyruvate kinase was shortened by 20 and 14 amino acids from the N- and C-terminus, leading to a final sequence length of 347 amino acids, which was comparable to the 339 amino acids in IgBP1. The simulation was supported with 14 experimental intra-protein cross-links (see Table S1).

#### Protein-protein docking

Protein complexes from the protein-protein docking benchmark dataset version 4.0 from the Weng lab [39] were used to compare the performance of *ab initio* docking and cross-link guided protein-protein docking. This benchmark data set features high-resolution protein complexes that are non-homologous and for which X-ray or NMR models in unbound conformations of the complex substituents exist. As docking methods struggle most with proteins that undergo large conformational changes upon association, we focused our performance analysis only on 16 binary protein complexes from the “medium difficult” and “difficult” category (see Table 3). Protein complexes in both categories show medium to large conformational changes at their interface with Interface Root Mean Square Deviations (I-RMSD) >1.5 and >2.2 Å, respectively, where the I-RMSD corresponds to the Cα RMSD of the interface residues after the unbound forms of the protein structures were superimposed on the bound form of the proteins [39].

**Table 3:** A selected list of 16 binary protein complexes from the “difficult” and “medium difficult” category of the protein docking benchmark dataset version 4.0 [39].

PDB-ID ChainId1:ChainId2	Difficulty class(M = Medium Difficulty,D = Difficult)	L-RMSD of best modelwithout XLs	Total number of virtualinter-protein XLs	Lowest L-RMSD amongthe 10 best modelsusing 7 randominter-protein XLs	L-RMSD of best modelusing 7 random inter-protein XLs
1MQ8 A:B	M	55.1	23	4.3	4.3
1JK9 A:B	D	40.3	31	6.8	7.2
2CFH A:C	M	38.2	16	7.1	7.6
2NZ8 A:B	M	24.1	35	7.9	7.9
1ATN A:D	D	44.7	9	8.0	8.0
1FQ1 A:B	D	31.5	28	8.0	8.0
1I2M A:B	M	30.9	25	8.4	8.4
2Z0E A:B	M	25.0	12	7.5	8.5
1F6M A:C	D	52.1	20	6.0	8.6
1BKD R:S	D	34.5	43	9.0	9.0
2J7P A:D	M	39.8	31	9.8	9.8
1HE8 B:A	M	90.8	38	10.7	10.7
1IBR A:B	D	47.3	49	13.0	13.0
1Y64 A:B	D	55.8	65	19.7	24.9
1IRA Y:X	D	35.7	26	34.4	34.4
1H1V A:G	D	83.0	20	42.4	42.4

Each complex has more than 7 predicted (virtual) inter-protein cross-links and was employed to test the impact of cross-links on protein docking calculations. Best models correspond to the models with the shortest mean SAS distance for all 7 cross-links. L-RMSD corresponds to the RMSD value among the smaller protein partners also known as ligands.

To assess the impact of the number of cross-links on docking calculations, predicted (virtual) inter-protein cross-link distances were calculated on the bound conformation of all 16 protein complexes using the *Xwalk* application. A virtual cross-link was assumed to form between a pair of lysine residues if the residue pair had an SAS distance ≤34.0 Å. The 34.0 Å threshold corresponds to the distance that around 80% of DSS and BS^3^ cross-links exhibit in a number of published XL-MS experiments (see section below). Among all virtual cross-links (see Table 3), one to seven were randomly chosen among 5 distance bins, namely 0–10 Å, 10–15 Å, 15–20 Å, 20–25 Å and 25–34 Å. The probability of choosing an inter-protein cross-link from a particular distance bin was predetermined by a probability distribution that was calculated on a large number of intra- and inter-protein cross-links from our new database, *XLdb* (see section below and Figure S2). The probabilities corresponded to 9%, 18%, 34%, 22% and 16% for the aforementioned distance bins. Given various lists of virtual cross-links for each of the 16 protein complexes, we next performed cross-link guided docking calculations to assess the impact of cross-links on the docking predictions.

The unbound conformations of the 16 protein pairs were first relaxed and subsequently docked at low-resolution (see steps 3.1.1 to 3.1.4). Around 100,000 docking models were generated for each protein pair and subsequently tested for their ability to satisfy one to seven virtual inter-protein cross-links. As all complexes had more than seven virtual cross-links (see Table 3), we generated 100 random selections for each number of cross-link. For each random selection, the model with the shortest mean distance was chosen as a best model for each protein complex. The quality of the predictions was assessed with the ligand RMSD (L-RMSD) measure, which corresponds to the Cα coordinate based RMSD between the smaller protein (ligand) in the “unbound complex” and in the predicted docking model after superposing them on the larger protein. Note that the L-RMSD value will always be notably larger than 0, as the unbound complexes in the difficult and medium difficult category show substantial atomic clashes between both binding partners.

### *XLdb*: A Database of Literature Curated Intra- and Inter-Protein Cross-links

We collected a non-comprehensive list of 506 intra-protein and 62 inter-protein cross-links from 14 recent publications. In the current form, all cross-links are stored in a database called *XLdb*, which is based on a Microsoft Excel sheet (Table S1). Figure S1, S2 and S3 provide frequency and probabilities plots for various distance ranges in *XLdb* (Text S1). Only cross-links that fulfill following criteria were included in the database: 1. XL-MS experiments must have been conducted with the DSS or BS^3^ cross-linking reagent. 2. Experimental structure on the cross-linked proteins must exist in the PDB. 3. The XL-MS data must have been published. *XLdb* is besides the recently published Xlink-DB [40] the only database that allows the mapping of cross-links on protein structures and testing of cross-link guided molecular modeling algorithms on a large scale. We hope that as a reference database, it will encourage new method developments in the field of data driven molecular modeling.

## Results

### Comparative Modeling for Cross-link Validation

**Figure 2:**
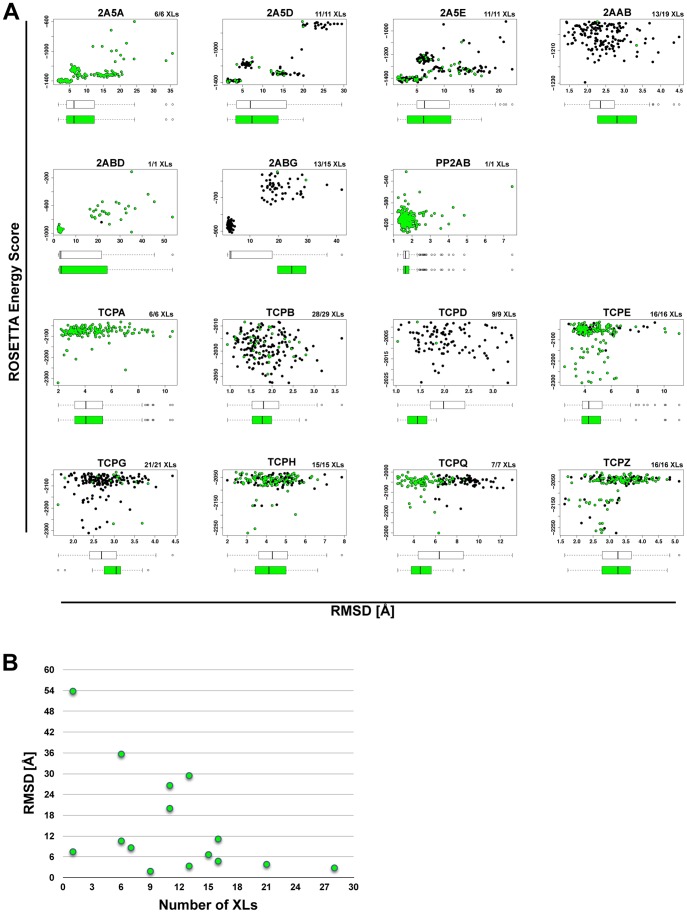
Comparative modeling calculations and chemical cross-link data validation on 15 proteins from the PP2A interaction network. (A) ROSETTA energy score versus RMSD plots for all proteins. Template structures (see Table 1) served as a reference for the RMSD calculations. Green colored dots highlight those models that satisfy most chemical cross-links; their numbers are indicated at the top right corner of each scatter plot. (B) For each protein from (A), only the model with the largest RMSD value is plotted demonstrating the prediction improvement with the increasing number of chemical cross-links.

We calculated comparative models using the workflow illustrated in Figure 1A for 15 proteins in the PP2A interaction network (Table 1) and 6 proteins from the reference data set (Table 2). Figure 2A shows the RMSD vs. *ROSETTA* energy scatter plots for all 15 PP2A proteins, where the RMSDs were calculated between the models and the template structure. The models satisfying most DSS based cross-links were furthermore highlighted in green. Two trends can be seen in the plots. First, models satisfying DSS cross-links can span a large RMSD range as evident from the box plots that are located below each scatter plot. There is, however, the tendency that the RMSD of the model with the lowest RMSD score, which nonetheless satisfies the largest number of cross-links, drops with increasing number of cross-links (see Figure 2B). Second, the model that satisfies the largest number of cross-links while being the closest to the template structure is in almost all cases among the top 3 models within the simulations (see Table 1). Thus, comparative modeling can be employed to validate XL-MS data while XL-MS data itself, in combination with a sophisticated scoring function, can aid in identifying native-like conformations. Similar conclusions can be drawn for the reference data set, where a similar range of RMSD values (with respect to their native structures) and rank positions for the best model satisfying most cross-links were observed (see Figure S4 and Table 2).

**Figure 3:**
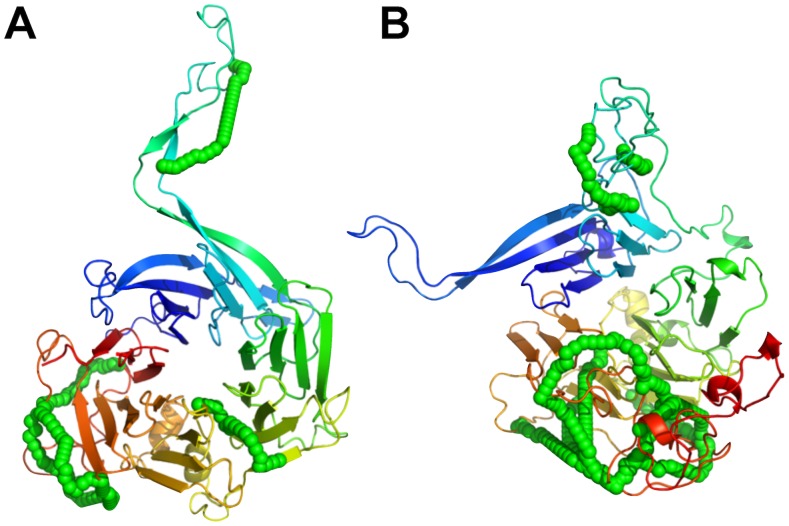
Chemical cross-links on the regulatory subunit 2ABG of PP2A might have originated from a stable intermediate folding state. (A) The comparative model that is most similar to its template structure 2ABA satisfies only 6 of 18 intra-protein cross-links. (B) In contrast, the comparative model that satisfies with 13 cross-links most of the cross-link data has a RMSD of 19.5 Å and is partially unfolded. Green chain of spheres indicate the shortest path between cross-linked lysine pairs that have an SAS distance ≤34.0 Å.

Two exceptions, namely TRFL_BOVIN and 2ABG_HUMAN showed large RMSD values of 8.5 Å and 19.6 Å, respectively, to their native or template structure. TRFL_BOVIN consists of two flexible C- and N-lobe domains [41]. As a result, some cross-links could have been formed on a conformation that is distinct from the one found in the PDB structure 1BLF. Interestingly 2ABG_HUMAN is a regulatory subunit of PP2A and has a WD40 propeller fold. It was found co-purified and cross-linked to the TRiC chaperonin complex as a substrate. We speculate therefore that some of the intra-protein cross-links stemmed from B regulatory subunits that were in a stable intermediate folding state while bound to the TRiC chaperonin complex. And indeed selecting for the lowest scoring model that satisfies most cross-links (13/18) shows only a partially folded WD40-propeller fold with an RMSD of 19.5 Å to the folded template structure (see Figure 3). Additional XL-MS experiments on affinity purified TRiC subunits that would provide a clean list of only intra-protein cross-links from 2ABG while in complex with TRiC, remain to be conducted.

### Cross-link Guided De Novo Modeling

**Figure 4:**
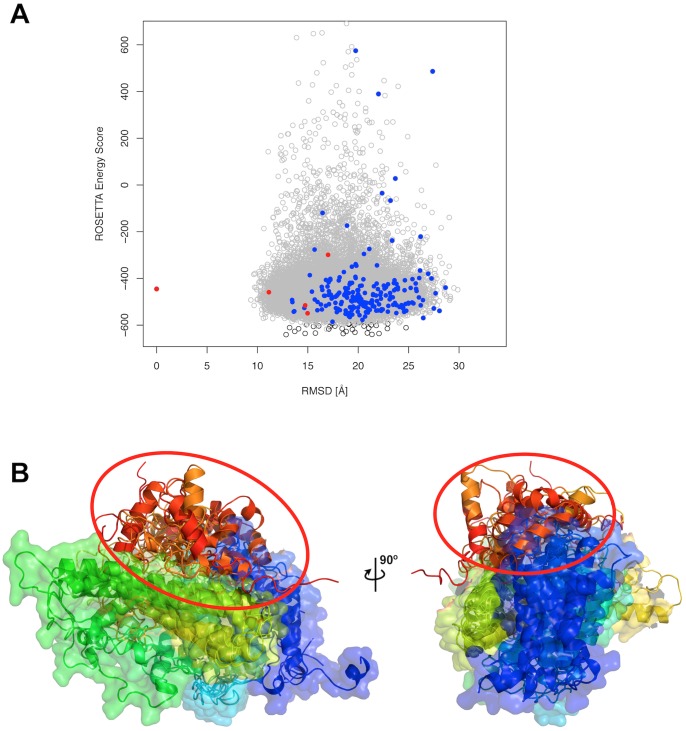
Localization of the C-terminal domain of IgBP1 with chemical cross-link data. (A) ***ROSETTA*** energy score versus RMSD plot for full-length models of IgBP1, with one of the best models acting as a reference structure for the RMSD calculation. Only models below an energy score of 650 are shown. Grey empty circles are models that satisfy more than 60 cross-links by Euclidean distance measure. Blue circles depict models that satisfy more than 60 cross-links by means of the SAS distance measure. The five red circles have been chosen as best models with RMSD ≤10.0 Å to the N-terminal template structure of mouse IgBP1 (PDB-ID: 3QC1). (B) Structure of the five best models. The structures are colored from blue to red between the N and C-terminus. The models were superimposed on their N-terminal domain highlighting the co-location of their C-terminal domain.

Immunoglobulin binding protein 1 (IgBP1) interacts with the catalytic subunit of PP2A, rendering it inactive and preventing it from proteasomal degradation [42]. As inter-protein chemical cross-links were found between IgBP1 and the catalytic subunit of PP2A, we constructed a partial *de novo* full-length model of human IgBP1 (see section De novo modeling) and predicted the interface between both proteins using cross-link guided protein-docking calculations (see section Protein-protein docking). For the partial *de* *novo* prediction of IgBP1, Cα distance restraints from the N-terminal region of the mouse homolog and 65 intra-protein cross-link distance restraints from our XL-MS experiments were applied [6]. Of the 65 intra-protein cross-links, 18 were found within the C-terminal region, while 32 were found between the N- and C-terminal domains of IgBP1. Crucial for the structure prediction was the application of *Xwalk*’s SAS distance as a post-processing filter. From around 157,000 structural models that were predicted, over 113,000 models satisfied at least 60 cross-links by means of the Euclidean distance. In contrast, only around 190 models satisfied the same number of cross-links using the SAS distance measure (see Figure 4A). Nevertheless, the structure prediction calculations did not converge as assessed by a RMSD based clustering attempt of the 190 models and thus did not result in an unambiguous fold prediction for the C-terminal domain (see Figure 4). However, the 32 intra-protein cross-links facilitated the localization of the C-terminal domain with respect to the N-terminal domain. Five models that had the lowest RMSD (≤10.0 Å) to the N-terminal domain of the template structure (PDB-ID: 3QC1) had their C-terminal domain co-localized at the same region (see Figure 4B). These five models were chosen as best models of IgBP1 and docked to the catalytic subunits of PP2A as described in the next section.

A similar structure prediction for the pyruvate kinase from the reference data set (see section Comparative and *de novo* modeling) with only 14 experimental intra-protein cross-links produced models with RMSD values down to 5.93 Å to the native structure while satisfying all 14 intra-protein cross-links. In contrary to the IgBP1 calculation, the simulation for the pyruvate kinase did converge, producing 5 clusters with at least 24 members, of which the lowest scoring models are shown in Figure S5. Thus, our workflow coupled with chemical cross-link data enables the prediction of useful structural information for large proteins with only partial structural data.

### Cross-link Guided Protein-protein Docking

We developed a protein-protein docking workflow [6] (see Figure 1C) and applied it to predict the conformation and the binding interface between the catalytic subunits of PP2A and its interactor IgBP1. For the docking calculations 7 inter-protein cross-links, 11 intra-protein cross-links and 10 mono-links were utilized to guide the calculations between PP2AA and IgBP1.

**Figure 5:**
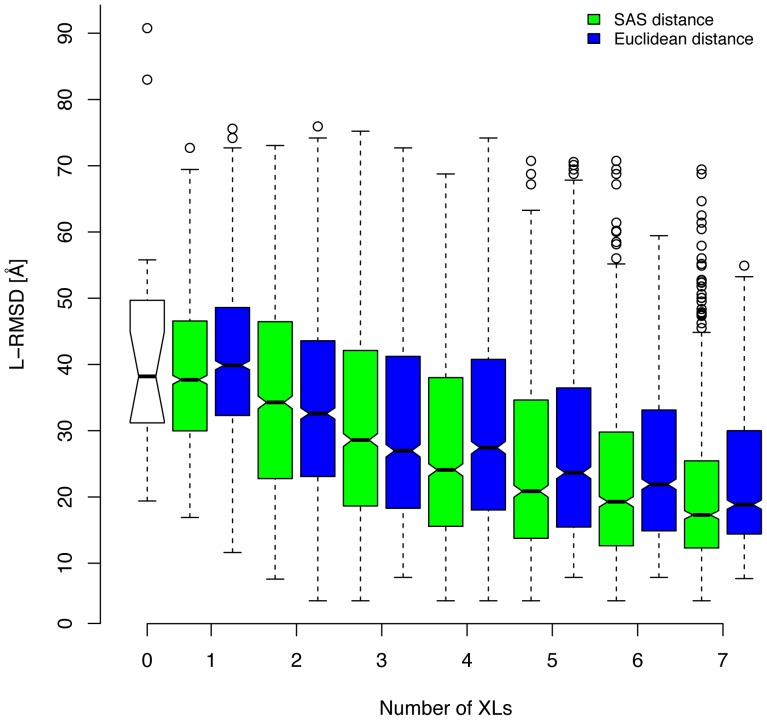
Box plots showing the improvement of the docking predictions with an increasing number of cross-links (XLs). The data was collected on 16 protein complexes that were docked using 100 random selections of 1 to 7 virtual cross-links. For each random selection the model satisfying all cross-links and having the shortest mean cross-link distance was selected and its ligand RMSD (L-RMSD) value selected for plotting. Distances were measured with the Solvent Accessible Surface (SAS) distance (green boxes) or the Euclidean distance (blue boxes). White box corresponds to blind docking without distance restraints.

Prior to the docking calculations, we first tested the impact of the number of cross-links on protein-protein docking calculations. For this purpose, 16 protein complexes with experimental structural coordinates in the PDB from a docking benchmark data set were docked and the impact of randomly chosen 1 to 7 inter-protein cross-links was assessed on the docking results (see section Reference data sets for the modeling workflows). The boxplots in Figure 5 show the docking performance of any model that satisfies a certain number of randomly selected virtual cross-links. The performance was assessed by the ligand’s RMSD values (L-RMSD). A clear trend towards higher quality predictions with increasing number of cross-links was observed. On average, the improvement in docking predictions with the SAS distance measure rose by 5 Å L-RMSD per cross-link and leveled off at 5 cross-links in total, which agreed with a similar observation made elsewhere [43]. The Euclidean distance measure had the same tendencies, although less pronounced and with higher median L-RMSD values as compared to the SAS distance measure.

**Figure 6:**
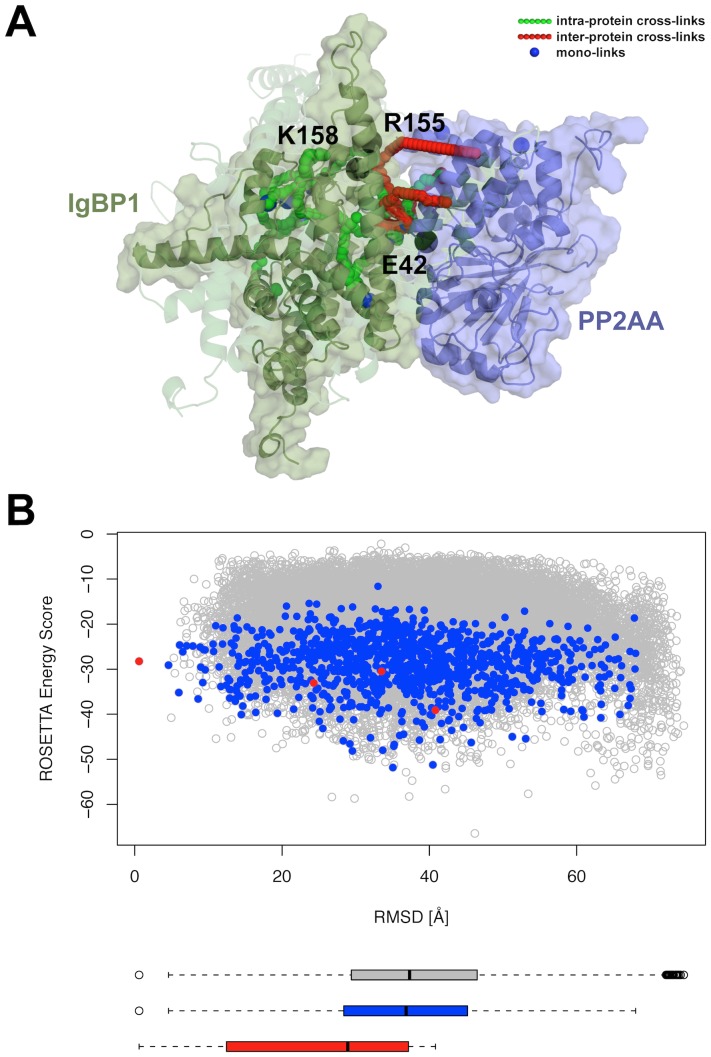
Prediction of the IgBP1-PP2AA protein topology using 7 inter-protein cross-links, 11 intra-protein cross-links and 10 mono-links. (A) Structural model of the lowest scoring models from the 4 largest clusters, showing the PP2AA protein in purple color and the IgBP1 protein in dark green color. The solid cartoon representation corresponds to the cluster representative of the largest cluster, while the transparent IgBP1 models are cluster representatives of the 2nd, 3rd and 4th largest cluster. Intra-links with their shortest SAS distance path are shown as green colored chains of spheres, inter-links are shown in red and mono-links are highlighted as blue spheres. In addition, black spheres indicate previously mutated amino acids that were shown to be involved in forming the interface of IgBP1 and PP2AA. (B) Overview of the *ROSETTA* energy scores for all models that satisfied at least 6 inter-protein cross-links by means of the Euclidean distance measure are shown as empty grey circles. The RMSD was calculated to the cluster representative of the largest cluster. Models satisfying at least 6 inter-protein cross-links by means of the SAS distance measure and having a binding interface size ≥900 Å^2^ are highlighted in blue, while the cluster representatives of the 4 largest clusters are highlighted as red colored circles.

The application of the cross-link guided docking protocol to the IgBP1-PP2AA protein complex (see steps 3.1.x) revealed 4 large clusters of predicted complex models. Despite the high L-RMSD values among the cluster representatives (see Figure 6B), all models revealed similar interface residues as highlighted by the similar location of IgBP1 with respect to PP2AA in Figure 6A. Three amino acids that had been shown in previous studies to form the interface can indeed be found at the interface of the cluster representatives (see Figure 6A).

## Discussion

Distance restraints derived from XL-MS experiments are useful for driving modeling calculations towards native-like conformations. In this manuscript, we have described three different computational protocols for comparative and *de novo* structure prediction and protein-protein docking and demonstrated the added value of the distance restraints on the computational predictions. Each workflow utilizes the *ROSETTA* molecular modeling suite at several steps in its calculation. The three workflows provide complementary predictions to other structure determination methods like X-ray crystallography and NMR spectroscopy. They have less restriction on the size and rigidity of proteins and are well suited to determine the topology of protein complexes. As XL-MS provides rather low-resolution information, it is particularly useful for gaining structural information on larger protein complexes. In addition, structural models of protein-protein binding interfaces can be generated in cases in which structural information of the subunits exist.

Protein-protein docking methods benefit most from XL-MS data. Native like conformations can be predicted even in cases in which homology models or *de novo* models for the binding partners are used. It is often possible to predict the topology of a complex with already two cross-links to an accuracy of less than 10 Å L-RMSD (see Figure 5). The increased accuracy in the docking predictions raises the question to which extent multimeric protein docking calculations could benefit from XL-MS data. This important question remains to be addressed.

A XL-MS experiment produces beside inter-protein cross-links also intra-protein cross-links and mono-links. However, due to the employment of the Euclidean distance measure as a mean to simulate cross-link data, the latter type of modifications have so far been mainly ignored and not been incorporated in docking calculations. *Xwalk,* however, allows exploiting these modifications under the assumption that intra-protein cross-links and mono-links are formed outside the protein-protein binding interface. Xwalk’s shortest path over the protein surface between modified amino acids mimics intra-and inter-protein cross-links. Models that display cross-links within the predicted binding interfaces can therefore be removed from further analysis. One drawback of *Xwalk* is its high computational expense when calculating SAS distances, which can take up to a second per cross-link. It would therefore be desirable to develop new, faster algorithms for simulating cross-links on protein surfaces. The faster algorithms could facilitate the inclusion of the SAS distance measure in scoring functions where they could directly impact conformational sampling routines rather than acting as a post-modeling filter.

It should be clear, however, that DSS based XL-MS data provide low-resolution structural information, which makes them less appropriate for fold prediction in comparative and *de novo* modeling. For example, despite using over 60 intra-protein cross-links for the full-length structure of IgBP1, we were unable to pinpoint the fold of the C-terminus. The main problem remains the difficulty to distinguish between close-native and nonnative conformations based purely on DSS cross-links, as it is apparent from the large RMSD ranges in Figure 2. At the same time, the low-resolution information might be sufficient to probe large conformational changes on proteins (see Figure 3). Important for the application of DSS based XL-MS data to structure prediction is that the structural features can be probed with a 34.0 Å long “distance ruler” (see section Reference data sets for the modeling workflows).

Chemical cross-links that cannot be mapped on experimental structures or high-quality comparative models could have emerged as a result of alternative protein conformations or false positive identifications. The former cause might be stimulated by experimental cross-link conditions such as buffer solution, pH, salt concentration, absence of ligands etc. that can be distinct from the conditions found in crystallization or NMR experiments. The different experimental conditions can lead to conformational changes of the proteins or induce even different oligomeric states that might result in dissatisfied cross-links on experimental X-ray structures. The second cause is likely less relevant as the false positive rate in cross-linking experiments is often found to be around 5% or lower [22]. We therefore believe that the main source of apparently not satisfying cross-links remains the conformational variability of proteins especially in solution, like in the case of 2ABG (see Figure 3).

In conclusion, we have introduced three computational workflows for XL-MS data driven structural modeling of proteins and protein complexes. In combination with available structural models of proteins, these workflows strengthen XL-MS as a complementary approach for gaining structural insights into protein complexes and generating testable predictions for biologically relevant protein-protein interactions. The type, quality and coverage of the restraints are likely to increase with the ongoing efforts of the mass spectrometry community to improve XL-MS technology. On the other hand, a better understanding of protein folding kinetics and interaction mechanisms as well as more sophisticated algorithms for simulating XL-MS data will likely improve the prediction accuracy of cross-link guided molecular modeling. Taken together, data-driven structural modeling of proteins and protein complexes as a hybrid structural biology method will likely have an increasing impact on the protein structure and modeling fields.

## Supporting Information

Figure S1 **Histogram of SAS distances as found in the cross-link database** ***XLdb*** **(see Table S1).**
(TIF)

Figure S2 **Probabilities for observing a cross-link between 0 and 34.0 Å SAS distance.** The probabilities were calculated with an empirical cumulative distribution function that was applied to all cross-links from the cross-link database *XLdb* (see Table S1) having a distance between 0 and 34 Å.
(TIF)

Figure S3 **Empirical cumulative distribution functions applied on the entire cross-link database** ***XLdb*** **(see Table S1).**
(TIF)

Figure S4 **Cross-Link Guided Comparative Modeling on a Benchmark Data Set.** The performance of the modeling calculations was assessed by the Cα RMSD similarity between the predicted models and the native protein structure (see Table 2). Green colored dots show those models that satisfy most chemical cross-links; their numbers are indicated at the top right corner of each scatter plot.
(TIF)

Figure S5 **Cross-Link Guided De Novo Modeling on the Benchmark Protein KCRM_RABIT.** (A) *ROSETTA* energy score versus RMSD plot for full-length models of KCRM_RABIT. Grey empty circles are all 105,294 models. Black circles depict models that satisfy all 14 intra-protein cross-links by means of the SAS distance measure. The five red circles are the lowest scoring models from the 5 largest clusters after clustering the lowest scoring 500 black circled models with a 10.0 Å RMSD cut-off. Compared to the five blue circles that represent the 5 largest clusters in a non-guided *de novo* prediction, the mean RMSD value drops from 12.5 Å to 9.7 Å. (B) Structure of the native KCRM_RABIT structure (PDB-ID: 1U6R) is shown on the left, while the five best models are shown on the right. The structures are colored from blue to red between the N and C-terminus. The de novo modeled N-terminal domain is encircled, while the C-terminal domain for which a template structure was provided is shown in transparent surface representation. Note the co-localization of the de novo modeled N-terminal domain.
(TIF)

Table S1 **List of cross-links in** ***XLdb*** **.**
(XLSX)

Text S1 ***XLdb*** **, a cross-link database.**
(DOCX)

Text S2 **Checklist for Cross-Link guided Protein-Protein Docking Demo.**
(TXT)

## References

[1] Zhang QC, Petrey D, Deng L, Qiang L, Shi Y, et al.. (2012) Structure-based prediction of protein–protein interactions on a genome-wide scale. Nature. doi:10.1038/nature11503. 10.1038/nature11503 PMC3482288 23023127

[2] Wang X, Wei X, Thijssen B, Das J, Lipkin SM, et al (2012) Three-dimensional reconstruction of protein networks provides insight into human genetic disease. Nat Biotechnol 30: 159–164 doi:10.1038/nbt.2106 22252508 10.1038/nbt.2106 PMC3708476

[3] Edwards A (2009) Large-Scale Structural Biology of the Human Proteome. Annu Rev Biochem 78: 541–568 doi:10.1146/annurev.biochem.78.070907.103305 19489729 10.1146/annurev.biochem.78.070907.103305

[4] Krissinel E, Henrick K (2007) Inference of macromolecular assemblies from crystalline state. J Mol Biol 372: 774–797. 17681537 10.1016/j.jmb.2007.05.022

[5] Mosca R, Pons C, Fernández-Recio J, Aloy P (2009) Pushing Structural Information into the Yeast Interactome by High-Throughput Protein Docking Experiments. PLoS Comput Biol 5: e1000490 doi:10.1371/journal.pcbi.1000490 19714207 10.1371/journal.pcbi.1000490 PMC2722787

[6] Herzog F, Kahraman A, Boehringer D, Mak R, Bracher A, et al (2012) Structural probing of a protein phosphatase 2A network by chemical cross-linking and mass spectrometry. Science 337: 1348–1352 doi:10.1126/science.1221483 22984071 10.1126/science.1221483

[7] Rappsilber J (2011) The beginning of a beautiful friendship: Cross-linking/mass spectrometry and modelling of proteins and multi-protein complexes. Journal of Structural biology 173: 530–540 doi:10.1016/j.jsb.2010.10.014 21029779 10.1016/j.jsb.2010.10.014 PMC3043253

[8] Chen ZA, Jawhari A, Fischer L, Buchen C, Tahir S, et al (2010) Architecture of the RNA polymerase II–TFIIF complex revealed by cross-linking and mass spectrometry. Embo J 29: 717–726 doi:10.1038/emboj.2009.401 20094031 10.1038/emboj.2009.401 PMC2810376

[9] Blattner C, Jennebach S, Herzog F, Mayer A, Cheung ACM, et al (2011) Molecular basis of Rrn3-regulated RNA polymerase I initiation and cell growth. Gene Dev 25: 2093–2105 doi:10.1101/gad.17363311 21940764 10.1101/gad.17363311 PMC3197207

[10] Bohn S, Beck F, Sakata E, Walzthoeni T, Beck M, et al (2010) Structure of the 26S proteasome from Schizosaccharomyces pombe at subnanometer resolution. Proc Natl Acad Sci U S A 107: 20992–20997 doi:10.1073/pnas.1015530107 21098295 10.1073/pnas.1015530107 PMC3000292

[11] Lasker K, Forster F, Bohn S, Walzthoeni T, Villa E, et al (2012) Molecular architecture of the 26S proteasome holocomplex determined by an integrative approach Vol. 109: 1380–1387. 10.1073/pnas.1120559109 PMC3277140 22307589

[12] Trnka MJ, Burlingame AL (2010) Topographic Studies of the GroEL-GroES Chaperonin Complex by Chemical Cross-linking Using Diformyl Ethynylbenzene. Mol Cell Proteomics 9: 2306–2317 doi:10.1074/mcp.M110.003764 20813910 10.1074/mcp.M110.003764 PMC2953922

[13] Kalisman N, Adams CM, Levitt M (2012) Subunit order of eukaryotic TRiC/CCT chaperonin by cross-linking, mass spectrometry, and combinatorial homology modeling. Proc Natl Acad Sci U S A. doi:10.1073/pnas.1119472109. 10.1073/pnas.1119472109 PMC3287007 22308438

[14] Leitner A, Joachimiak LA, Bracher A, Mönkemeyer L, Walzthoeni T, et al.. (2012) The Molecular Architecture of the Eukaryotic Chaperonin TRiC/CCT. Structure/Folding and Design: 1–12. doi:10.1016/j.str.2012.03.007. 10.1016/j.str.2012.03.007 PMC3350567 22503819

[15] Mädler S, Bich C, Touboul D, Zenobi R (2009) Chemical cross-linking with NHS esters: a systematic study on amino acid reactivities. J Mass Spectrom 44: 694–706 doi:10.1002/jms.1544 19132714 10.1002/jms.1544

[16] Leitner A, Walzthoeni T, Kahraman A, Herzog F, Rinner O, et al (2010) Probing native protein structures by chemical cross-linking, mass spectrometry, and bioinformatics. Molecular & cellular proteomics : MCP 9: 1634–1649 doi:10.1074/mcp.R000001-MCP201 20360032 10.1074/mcp.R000001-MCP201 PMC2938055

[17] Mayne SLN, Patterton H-G (2011) Bioinformatics tools for the structural elucidation of multi-subunit protein complexes by mass spectrometric analysis of protein-protein cross-links. Brief Bioinform 12: 660–671 doi:10.1093/bib/bbq087 22101029 10.1093/bib/bbq087

[18] Leitner A, Reischl R, Walzthoeni T, Herzog F, Bohn S, et al (2012) Expanding the chemical cross-linking toolbox by the use of multiple proteases and enrichment by size exclusion chromatography. Molecular & cellular proteomics : MCP 11: M111.014126 doi:10.1074/mcp.M111.014126 10.1074/mcp.M111.014126 PMC3316732 22286754

[19] Luo J, Fishburn J, Hahn S, Ranish J (2012) An integrated chemical cross-linking and mass spectrometry approach to study protein complex architecture and function. Molecular & cellular proteomics : MCP 11: M111.008318 doi:10.1074/mcp.M111.008318 10.1074/mcp.M111.008318 PMC3277749 22067100

[20] Paramelle D, Miralles G, Subra G, Martinez J (2013) Chemical cross-linkers for protein structure studies by mass spectrometry. Proteomics. doi:10.1002/pmic.201200305. 10.1002/pmic.201200305 23255214

[21] Yang B, Wu Y-J, Zhu M, Fan S-B, Lin J, et al (2012) Identification of cross-linked peptides from complex samples. Nat Methods 9: 904–906 doi:10.1038/nmeth.2099 22772728 10.1038/nmeth.2099

[22] Walzthoeni T, Claassen M, Leitner A, Herzog F, Bohn S, et al (2012) False discovery rate estimation for cross-linked peptides identified by mass spectrometry. Nat Methods 9: 901–903 doi:doi:10.1038/nmeth.2103 22772729 10.1038/nmeth.2103

[23] Green N, Reisler E, Houk K (2001) Quantitative evaluation of the lengths of homobifunctional protein cross-linking reagents used as molecular rulers. Protein Sci 10: 1293–1304. 11420431 10.1110/ps.51201 PMC2374107

[24] Potluri S, Khan AA, Kuzminykh A, Bujnicki JM, Friedman AM, et al (2004) Geometric analysis of cross-linkability for protein fold discrimination. Pac Symp Biocomput 9: 447–458. 10.1142/9789812704856_0042 14992524

[25] Zelter A, Hoopmann MR, Vernon R, Baker D, MacCoss MJ, et al (2010) Isotope Signatures Allow Identification of Chemically Cross-Linked Peptides by Mass Spectrometry: A Novel Method to Determine Interresidue Distances in Protein Structures through Cross-Linking. J Proteome Res 9: 3583–3589 doi:10.1021/pr1001115 20476776 10.1021/pr1001115 PMC2917471

[26] Kahraman A, Malmström L, Aebersold R (2011) Xwalk: computing and visualizing distances in cross-linking experiments. Bioinformatics 27: 2163–2164 doi:10.1093/bioinformatics/btr348 21666267 10.1093/bioinformatics/btr348 PMC3137222

[27] Söding J, Biegert A (2005) The HHpred interactive server for protein homology detection and structure prediction. Nucleic Acids Res. 10.1093/nar/gki408 PMC1160169 15980461

[28] Rice P, Longden I, Bleasby A (2000) EMBOSS: The European molecular biology open software suite. Trends Genet 16: 276–277. 10827456 10.1016/s0168-9525(00)02024-2

[29] Kim D, Chivian D, Baker D (2004) Protein structure prediction and analysis using the Robetta server. Nucleic Acids Res 32: W526–W531 doi:10.1093/nar/gkh468 15215442 10.1093/nar/gkh468 PMC441606

[30] Jones D (1999) Protein secondary structure prediction based on position-specific scoring matrices 10.1006/jmbi.1999.3091 : Journal of Molecular Biology | ScienceDirect.com. J Mol Biol. 10.1006/jmbi.1999.3091 10493868

[31] Berman H, Westbrook J, Feng Z, Gilliland G, Bhat T, et al (2000) The Protein Data Bank. Nucleic Acids Res 28: 235–242. 10592235 10.1093/nar/28.1.235 PMC102472

[32] Team RDC (2010) R: A Language and Environment for Statistical Computing. Vienna, Austria: R Foundation for Statistical Computing. Available: http://www.R-project.org.

[33] Hubbard S, Thornton J (1993) Naccess. Department of Biochemistry and Molecular Biology, University College London: Computer Program.

[34] Janin J, Bahadur RP, Chakrabarti P (2008) Protein-protein interaction and quaternary structure. Q Rev Biophys 41: 133–180 doi:10.1017/S0033583508004708 18812015 10.1017/S0033583508004708

[35] Kahraman A, Morris RJ, Laskowski RA, Thornton JM (2007) Shape variation in protein binding pockets and their ligands. J Mol Biol 368: 283–301 doi:10.1016/j.jmb.2007.01.086 17337005 10.1016/j.jmb.2007.01.086

[36] Kahraman A, Morris RJ, Laskowski RA, Favia AD, Thornton JM (2010) On the diversity of physicochemical environments experienced by identical ligands in binding pockets of unrelated proteins. Proteins 78: 1120–1136 doi:10.1002/prot.22633 19927322 10.1002/prot.22633

[37] Gray J, Moughon S, Wang C, Schueler-Furman O, Kuhlman B, et al (2003) Protein-protein docking with simultaneous optimization of rigid-body displacement and side-chain conformations. J Mol Biol 331: 281–299 doi:10.1016/S0022-2836(03)00670-3 12875852 10.1016/s0022-2836(03)00670-3

[38] Punta M, Coggill PC, Eberhardt RY, Mistry J, Tate J, et al (2012) The Pfam protein families database. Nucleic Acids Res 40: D290–D301 doi:10.1093/nar/gkr1065 22127870 10.1093/nar/gkr1065 PMC3245129

[39] Hwang H, Vreven T, Janin J, Weng Z (2010) Protein-protein docking benchmark version 4.0. Proteins 78: 3111–3114 doi:10.1002/prot.22830 20806234 10.1002/prot.22830 PMC2958056

[40] Zheng C, Weisbrod CR, Chavez JD, Eng JK, Sharma V, et al (2013) XLink-DB: Database and Software Tools for Storing and Visualizing Protein Interaction Topology Data. J Proteome Res 12: 1989–1995 doi:10.1021/pr301162j 23413830 10.1021/pr301162j PMC3744611

[41] Baker EN, Anderson BF, Baker HM, Haridas M, Jameson GB, et al (1991) Structure, function and flexibility of human lactoferrin. International Journal of Biological Macromolecules 13: 122–129. 1911553 10.1016/0141-8130(91)90036-t

[42] Prickett T, Brautigan D (2004) Overlapping binding sites in protein phosphatase 2A for association with regulatory A and alpha-4 (mTap42) subunits. J Biol Chem 279: 38912–38920 doi:10.1074/jbc.M401444200 15252037 10.1074/jbc.M401444200

[43] Shih ESC, Hwang M-J (2012) On the use of distance constraints in protein-protein docking computations. Proteins 80: 194–205 doi:10.1002/prot.23179 22038781 10.1002/prot.23179

[44] DeLano W (2002) The PyMOL Molecular Graphics System.

