# Supplementary figures and images for: Cross-Link Guided Molecular Modeling with ROSETTA
Source: PLoS One. 2013 Sep 17;8(9):e73411. doi: 10.1371/journal.pone.0073411 (PMC3775805; doi:10.1371/journal.pone.0073411)

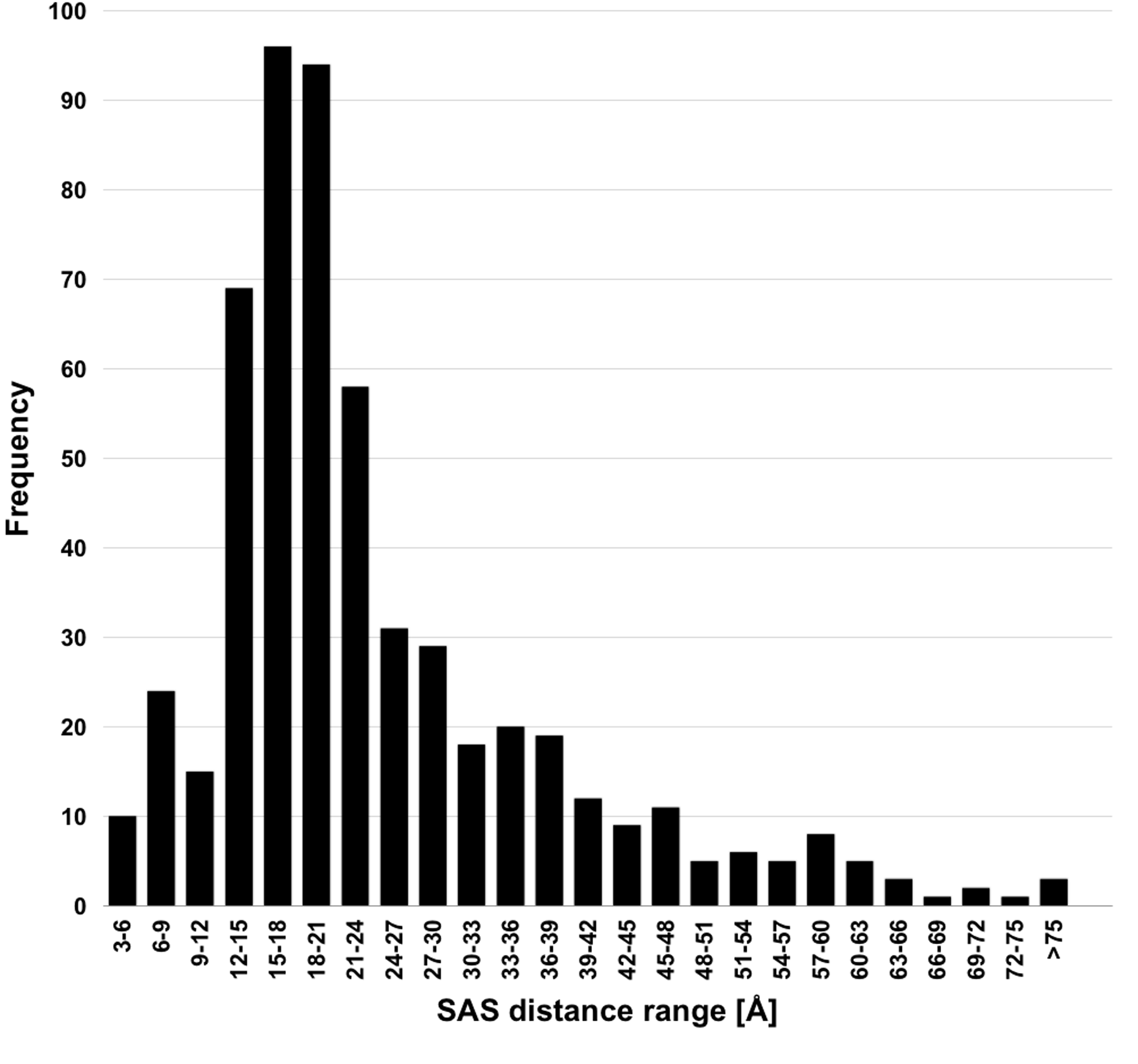

Supplement: Figure S1 — Histogram of SAS distances as found in the cross-link database XLdb (see Table S1). (TIF) [file pone.0073411.s001.tif]

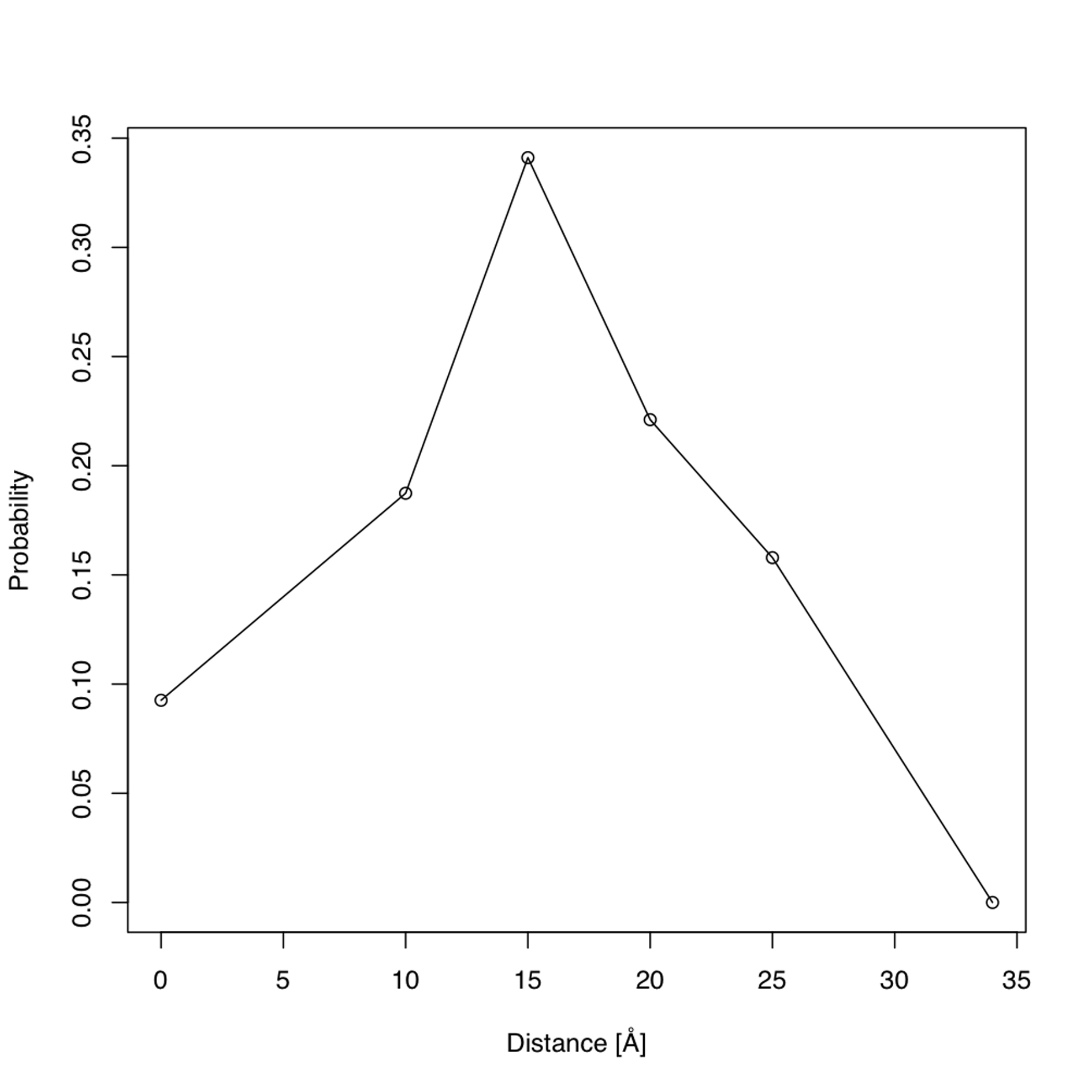

Supplement: Figure S2 — Probabilities for observing a cross-link between 0 and 34.0 Å SAS distance. The probabilities were calculated with an empirical cumulative distribution function that was applied to all cross-links from the cross-link database XLdb (see Table S1) having a distance between 0 and 34 Å. (TIF) [file pone.0073411.s002.tif]

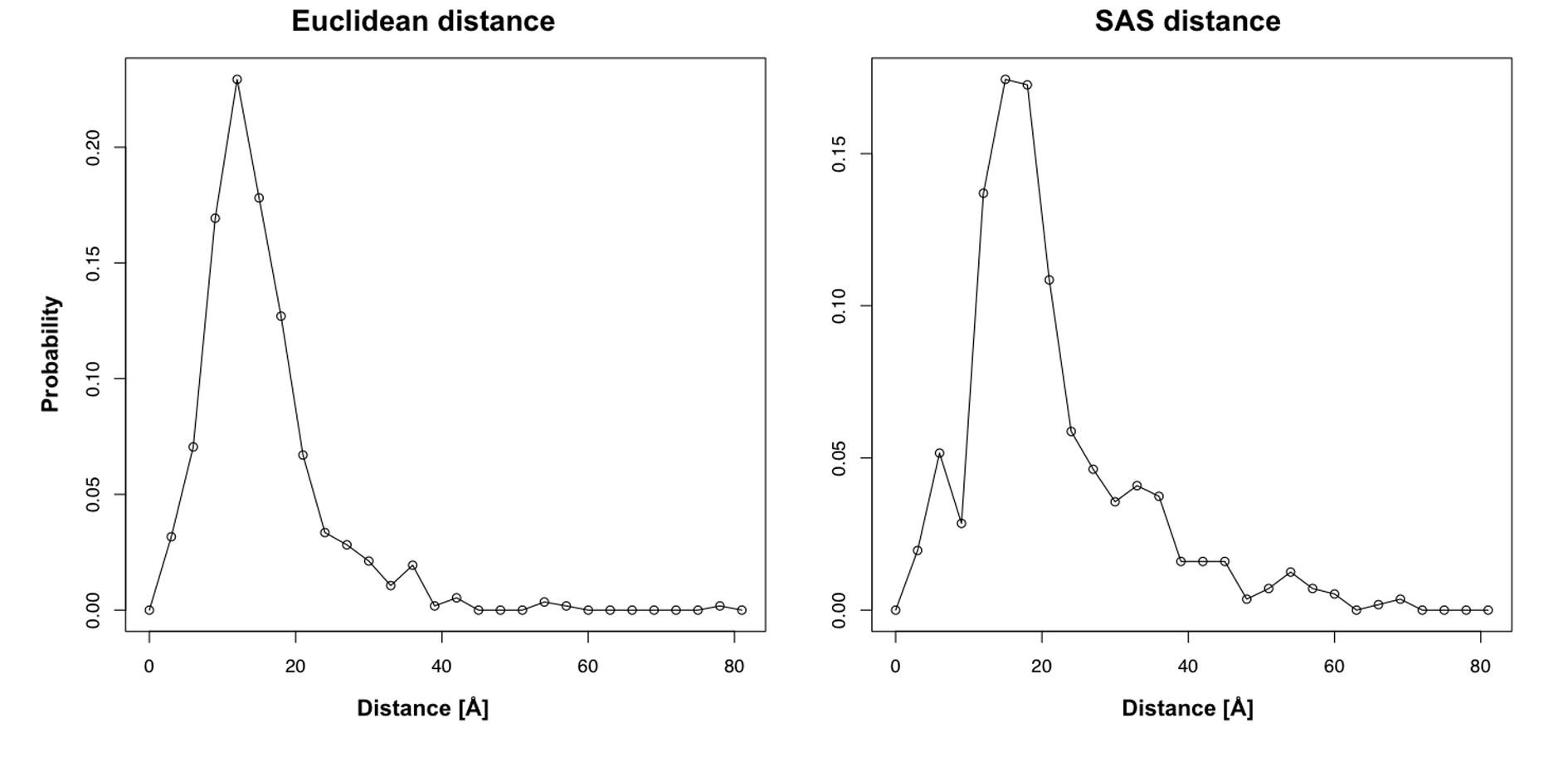

Supplement: Figure S3 — Empirical cumulative distribution functions applied on the entire cross-link database XLdb (see Table S1). (TIF) [file pone.0073411.s003.tif]

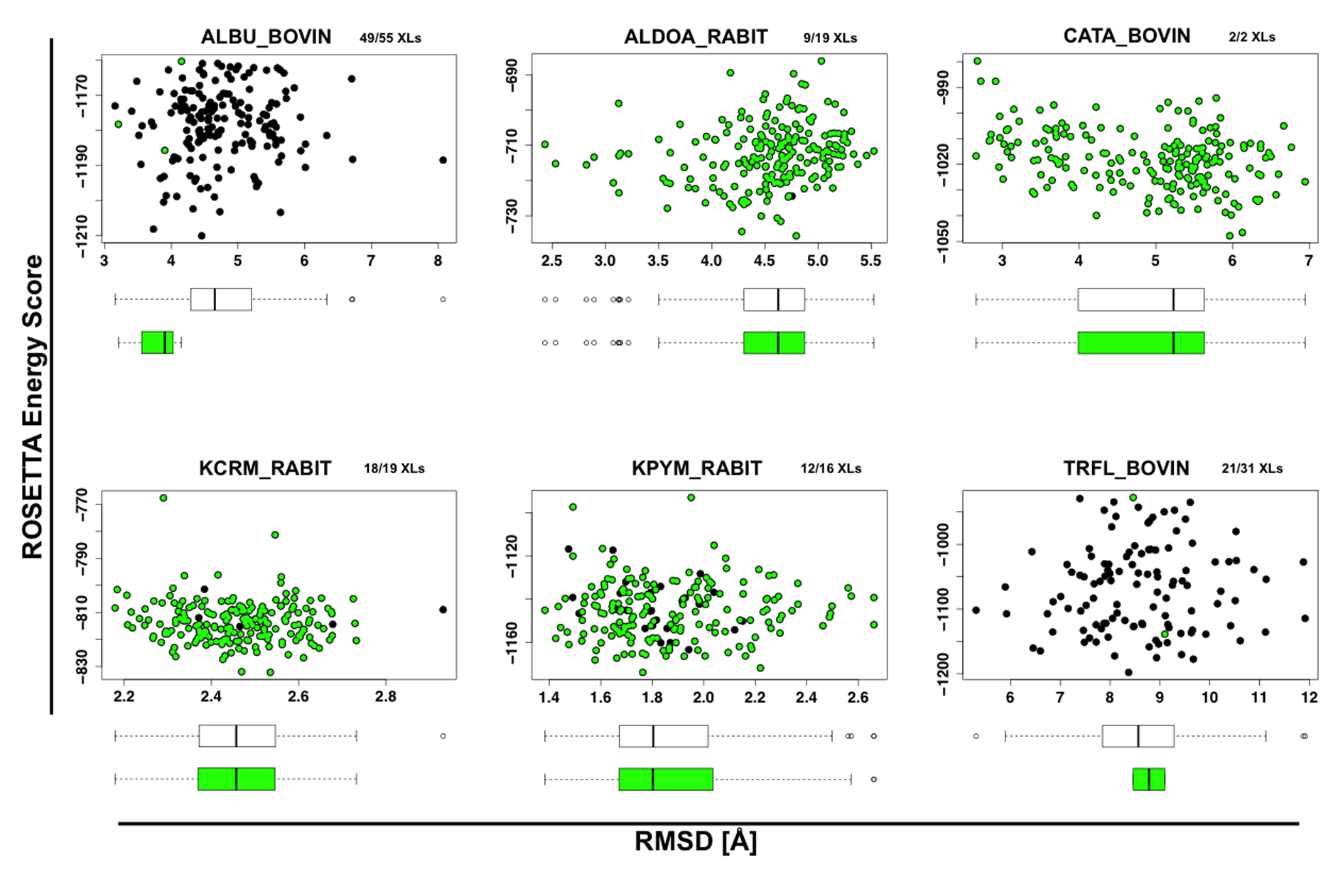

Supplement: Figure S4 — Cross-Link Guided Comparative Modeling on a Benchmark Data Set. The performance of the modeling calculations was assessed by the Cα RMSD similarity between the predicted models and the native protein structure (see Table 2). Green colored dots show those models that satisfy most chemical cross-links; their numbers are indicated at the top right corner of each scatter plot. (TIF) [file pone.0073411.s004.tif]

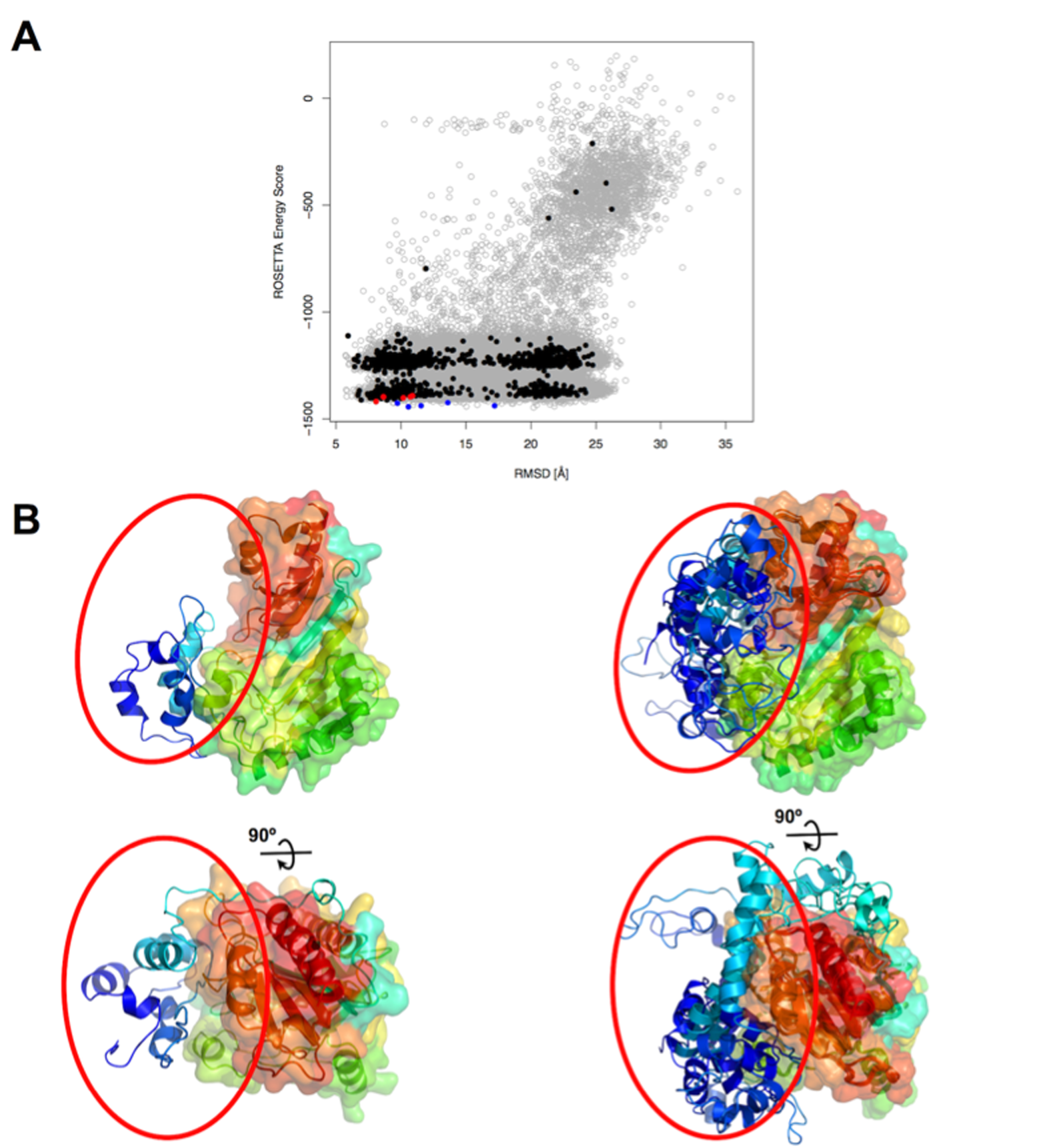

Supplement: Figure S5 — Cross-Link Guided De Novo Modeling on the Benchmark Protein KCRM_RABIT. (A) ROSETTA energy score versus RMSD plot for full-length models of KCRM_RABIT. Grey empty circles are all 105,294 models. Black circles depict models that satisfy all 14 intra-protein cross-links by means of the SAS distance measure. The five red circles are the lowest scoring models from the 5 largest clusters after clustering the lowest scoring 500 black circled models with a 10.0 Å RMSD cut-off. Compared to the five blue circles that represent the 5 largest clusters in a non-guided de novo prediction, the mean RMSD value drops from 12.5 Å to 9.7 Å. (B) Structure of the native KCRM_RABIT structure (PDB-ID: 1U6R) is shown on the left, while the five best models are shown on the right. The structures are colored from blue to red between the N and C-terminus. The de novo modeled N-terminal domain is encircled, while the C-terminal domain for which a template structure was provided is shown in transparent surface representation. Note the co-localization of the de novo modeled N-terminal domain. (TIF) [file pone.0073411.s005.tif]
