# Supplementary material for: Cross-Link Guided Molecular Modeling with ROSETTA
Source: PLoS One. 2013 Sep 17;8(9):e73411. doi: 10.1371/journal.pone.0073411 (PMC3775805; doi:10.1371/journal.pone.0073411)
Supplement: Text S1 — XLdb , a cross-link database. (DOCX) [file pone.0073411.s007.docx]

1. *XLdb*, a cross-link database

Chemical cross-linking combined with mass-spectrometry (XL-MS) data from 14 different publications [1-14] were collected into a Microsoft Excel sheet “database” (see Table S1). The database encompasses a total number of 506 intra-protein and 62 inter-protein cross-links that were all generated with the disuccinimidyl suberate (DSS) or bis(sulfosuccinimidyl) suberate (BS^3^) cross-linker reagent. The most important feature of the cross-link database is that experimental structural data in the Protein Data Bank (PDB) exists for all cross-linked proteins. The structural data permits the mapping of each chemical cross-link on a protein structure, which is essential for distance measurements of XL-MS data (see Table S1).

We performed a Wilcoxon rank sum test on the SAS distances of the intra-protein and inter-protein cross-links and observed that both distance distributions are similar with a p-value of 0.98. The similarity allowed the joining of both distances distributions into a single distribution for which the histogram is presented in Figure S1. At the same time 443 selected cross-links with SAS distances ≤ 34.0 Å were employed to calculate probabilities for observing a cross-links at a specific distance range (see Figure S2). The probabilities were used to bias the selection of virtual cross-links in the benchmark dataset (see Figure 5). Similar probabilities for the entire database were calculated and included as probabilities into the Xwalk application, version 0.6 (see Figure S3).

It should be noted that cross-linked amino acid pairs having a SAS distance > 34.0 Å in Table S1 cannot necessarily be regarded as false positive cross-link identifications. The structural data from X-ray crystallography or NMR experiments that were used for distance measurements might have been collected under different experimental conditions. Changes in the protein concentration, buffer solution, pH, salt concentration, absence of ligands etc. can cause conformational changes, which might explain longer cross-link distances. In addition, unknown oligomeric states of proteins might exist during cross-link experiments giving rise to inter-protein cross-links that due to the homomeric state of a protein complex were identified as intra-protein cross-links. It should also be pointed out, that Table S1 does not allow any judgment on the quality of a cross-link research project by the number of its satisfied cross-links. Research groups might have removed cross-links exceeding the distance threshold as false positives prior to publication, while others might have published all identified cross-links. In summary, Table S1 should not be used to evaluate research groups but rather should provide the modelling community a genuine and non-comprehensive list of cross-link data for assessing the difficulties of cross-link guided modeling calculations. We hope that in turn it will induce the development of new algorithms for cross-link guided molecular modeling.

References

1. Chen ZA, Jawhari A, Fischer L, Buchen C, Tahir S, et al. (2010) Architecture of the RNA polymerase II–TFIIF complex revealed by cross-linking and mass spectrometry. Embo J 29: 717–726. doi:10.1038/emboj.2009.401.

2. Herzog F, Kahraman A, Boehringer D, Mak R, Bracher A, et al. (2012) Structural probing of a protein phosphatase 2A network by chemical cross-linking and mass spectrometry. Science 337: 1348–1352. doi:10.1126/science.1221483.

3. Huang B, Dass C, Kim H (2005) Probing conformational changes of human serum albumin due to unsaturated fatty acid binding by chemical cross-linking and mass spectrometry. Biochem J 387: 695–702. doi:10.1042/BJ20041624.

4. Jacobsen R, Sale K, Ayson M, Novak P, Hong J, et al. (2006) Structure and dynamics of dark-state bovine rhodopsin revealed by chemical cross-linking and high-resolution mass spectrometry. Protein Sci 15: 1303–1317. doi:10.1110/ps.052040406.

5. Kalkhof S, Ihling C, Mechtler K, Sinz A (2005) Chemical cross-linking and high-performance Fourier transform ion cyclotron resonance mass spectrometry for protein interaction analysis: Application to a calmodulin/target peptide complex. Anal Chem 77: 495–503. doi:10.1021/ac0487294.

6. Lauber MA, Reilly JP (2010) Novel Amidinating Cross-Linker for Facilitating Analyses of Protein Structures and Interactions. Anal Chem 82: 7736–7743. doi:10.1021/ac101586z.

7. Lee YJ, Lackner LL, Nunnari JM, Phinney BS (2007) Shotgun Cross-Linking Analysis for Studying Quaternary and Tertiary Protein Structures. J Proteome Res 6: 3908–3917. doi:10.1021/pr070234i.

8. Mak E (2006) Using Chemical Crosslinking and Mass Spectrometry for Protein Model Validation and Fold Recognition University of Waterloo.

9. Mueller MQ, de Koning LJ, Schmidt A, Ihling C, Syha Y, et al. (2009) An Innovative Method To Study Target Protein-Drug Interactions by Mass Spectrometry. J Med Chem 52: 2875–2879. doi:10.1021/jm9000665.

10. Rinner O, Seebacher J, Walzthoeni T, Mueller L, Beck M, et al. (2008) Identification of cross-linked peptides from large sequence databases. Nat Methods 5: 315–318. doi:10.1038/nmeth.1192.

11. Seebacher J, Mallick P, Zhang N, Eddes JS, Aebersold R, et al. (2006) Protein Cross-Linking Analysis Using Mass Spectrometry, Isotope-Coded Cross-Linkers, and Integrated Computational Data Processing. J Proteome Res 5: 2270–2282. doi:10.1021/pr060154z.

12. Young M, Tang N, Hempel J, Oshiro C, Taylor E, et al. (2000) High throughput protein fold identification by using experimental constraints derived from intramolecular cross-links and mass spectrometry. P Natl Acad Sci Usa 97: 5802–5806.

13. Zelter A, Hoopmann MR, Vernon R, Baker D, MacCoss MJ, et al. (2010) Isotope Signatures Allow Identification of Chemically Cross-Linked Peptides by Mass Spectrometry: A Novel Method to Determine Interresidue Distances in Protein Structures through Cross-Linking. J Proteome Res 9: 3583–3589. doi:10.1021/pr1001115.

14. Leitner A, Reischl R, Walzthoeni T, Herzog F, Bohn S, et al. (2012) Expanding the chemical cross-linking toolbox by the use of multiple proteases and enrichment by size exclusion chromatography. Molecular & cellular proteomics : MCP 11: M111.014126. doi:10.1074/mcp.M111.014126.
